# Using a nasoseptal flap for the reconstruction of osteoradionecrosis in nasopharyngeal carcinoma: a case report

**DOI:** 10.1186/s40463-016-0139-1

**Published:** 2016-04-27

**Authors:** Mohamad Adel, Kai-Ping Chang

**Affiliations:** Department of Otolaryngology - Head & Neck Surgery, Chang Gung Memorial Hospital, No. 5, Fu-Hsing Street, Kwei-Shan, Tao-Yuan 33305 Taiwan; Department of Surgery, Al-Azhar University Hospitals in Cairo, Cairo, Egypt; College of Medicine, Chang Gung University, 259 Wen-Hua 1st Road, Kwei-Shan, Tao-Yuan 33302 Taiwan

**Keywords:** Osteoradionecrosis, Nasopharyngeal carcinoma, Radiotherapy, Nasoseptal flap, Skull base reconstruction

## Abstract

**Background:**

Osteoradionecrosis (ORN) is a challenging complication in patients with postirradiated nasopharyngeal carcinoma (NPC). The aim of this study was to evaluate the usage of a nasoseptal flap (NSF) after endoscopic sequestrectomy to reconstruct resultant skull base defects for the treatment of ORN.

**Case Presentation:**

We present our experience on the adaption of an NSF as a reconstruction method for bony clival defects after endoscopic sequestrectomy in patients with postirradiated NPC. We propose that by which the patient may be offered better and prompt surgical results in the more adequate coverage of a vascularized flap for the exposed defect and attenuate the hypoxic and inflammatory process among the NPC patients suffering from postirradiation ORN.

**Conclusions:**

To our knowledge, NSF use in such an indication has not been previously reported. Consequently, we advocate its utility for the management of patients with postirradiated skull base ORN in NPC in the future.

## Background

Nasopharyngeal carcinoma (NPC) is a rare head and neck cancer occurring in a surgically obscure and anatomically complex region of the body. Because of difficulty experienced accessing this region surgically, radiotherapy and concurrent chemoradiotherapy are treatment mainstays [1]. Although good disease control and long-term survival can be achieved in contemporary medical treatment, some potential complications still impair quality of life in patients with NPC. Long-term complications are diverse and include neuroendocrine and auditory complications, cranial nerve palsies, dry mouth, and osteoradionecrosis (ORN) of the skull base or cervical spine [2]. As the nasopharynx is anatomically obscure and difficult to examine in clinical practice, skull base ORN remains one of the most challenging complications of radiotherapy to diagnose and manage. Clinical diagnosis of skull base ORN is based on symptoms, imaging, and endoscopy. Clinical presentations of skull base ORN include rhinorrhea, a foul odor, a crust, epistaxis, and a persistent headache. Diagnosis is based on symptoms, imaging, and endoscopy. The pathological characteristics of ORN include fragments of dead bone and fibrotic tissue with chronic inflammation. Endoscopic evaluation of skull base ORN in postirradiated NPC reveals exposed necrotic bone and a copious crust in the nasopharynx [2].

Since we first described the management of skull base ORN in patients with postirradiated NPC in 2000, endoscopic sequestrectomy has become standard treatment [2]. Post-sequestrectomy wounds have typically been left to heal by secondary intention. Herein, we will report our experience treating a case of postirradiation skull base ORN using endoscopic sequestrectomy and an immediate nasoseptal flap (NSF) reconstruction for the resultant skull base defect.

## Case presentation

**Fig. 1:**
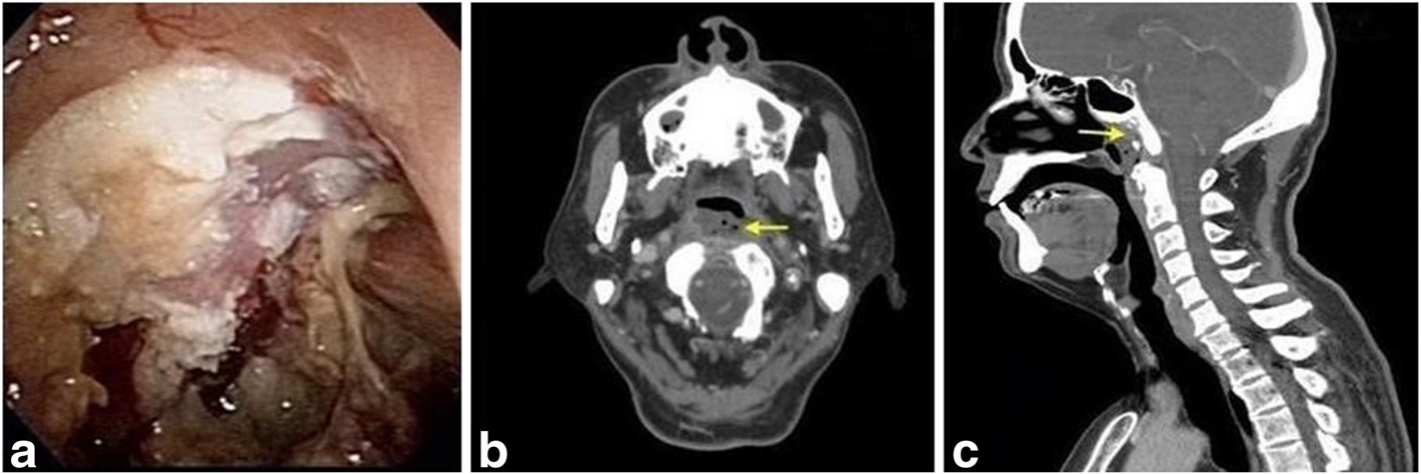
**a** Nasopharyngoscope shows crusts and pus accumulated on the nasopharynx, and sequestration of the skull base as a result of osteoradionecrosis. **b** & **c** CT of head and neck, axial and sagittal cuts, arrows show evidence of osteoradionecrosis of the skull base

A 65-year-old male with a past history of NPC, for which he was diagnosed as T3N1M0 and treated 32 years ago with radiotherapy, presented to our outpatient clinic complaining of a foul odor, purulent rhinorrhea, and occasional epistaxis. He also complained of some progressive symptoms over the preceding 2 months, such as bilateral tinnitus, a persistent headache, neck pain, dry mouth, and difficult swallowing. Nasopharyngoscopy showed an accumulation of thick crusts and necrotic bone over the roof of the nasopharynx (Fig. 1a).

**Fig. 2:**
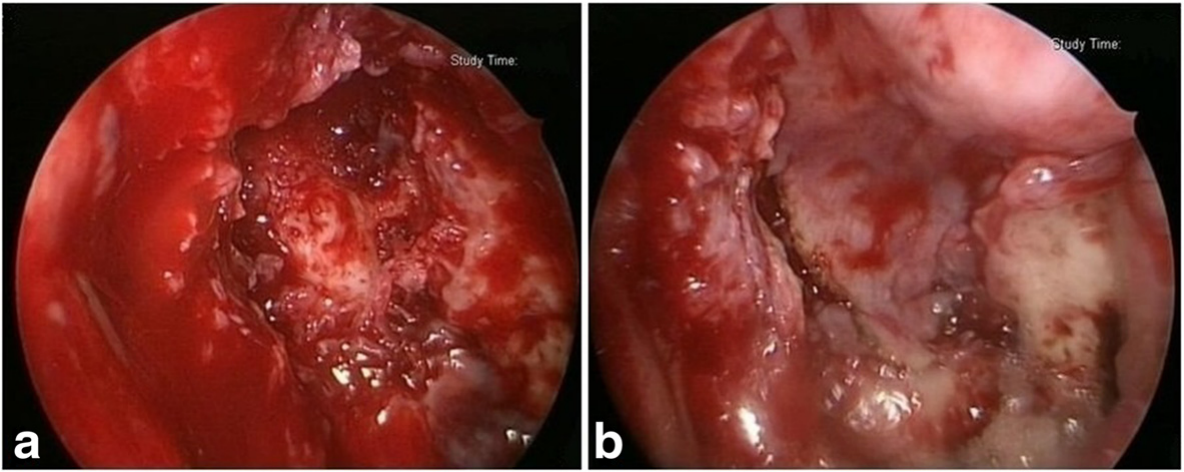
Endoscopic approach: **a** the resultant skull base defect after sequestrectomy. **b** Adaption of the nasoseptal flap to reconstruct the defect after sequestrectomy

A computed tomography scan of the head and neck revealed evidence of bone destruction with sclerotic changes of the clivus and necrotic soft tissue of the nasopharynx (Fig. 1b and c). With a presumed diagnosis of skull base ORN, endoscopic debridement and an immediate NSF reconstruction were performed. A powered burr was used to debride devitalized tissue and skull base bone, including the clivus. A vascularized NSF was harvested. A sphenoidotomy was performed, and the partial floor of the sphenoid sinus was removed to facilitate the removal of necrosis and flap transposition. A frozen-section biopsy revealed only chronic inflammation and fibrosis without any malignant cells. The flap was then used to cover the clivus and sphenoid floor defect (Fig. 2a and b). The flap was compressed with a nasal dressing (Nasopore Forte™, Polyganics, Groningen, Netherlands). A pathological examination of the removed specimens revealed necrotic bone and debris, without recurrent malignancy.

## Discussion

The pathophysiology of ORN development is complex. Suggested explanations for its development as a side effect of radiation are hypoxia, hypocellularity, and hypovascularity of the nasopharynx and adjacent skull base [3]. Additionally, mucosal changes and ciliary dysfunction resulting from radiotherapy might cause mucous and crusts accumulating over the inflamed mucosa [3]. Adding the effect of microorganisms, severe nasopharyngitis persists and causes additional inflammation, sequestration, and erosion of the underlying cortex [4]. If left untreated, these sequelae may lead to more serious and potentially life-threatening problems such as skull base ORN, optic neuritis, meningitis, and carotid blowout [1].

Clinically, ORN usually has a delayed and subtle presentation, with a latency period of more than 8 years after the initial treatment [5]. The latency period might be helpful in distinguishing it clinically from recurrent disease. However, it is important to exclude some radiation-induced malignancies, such as sarcoma [6]. If ORN presented earlier in the disease course, tumor recurrence would need to be excluded before performing any definitive management. If tumor recurrence is a concern for clinicians, NSF reconstruction should be delayed until a pathological report is available to prevent delaying diagnosis because of flap coverage. In our previous work, we advocated for the use of a molecular method using Epstein-Barr virus latent membrane protein-1 as a genetic marker to differentiate NPC recurrence from ORN [7]. The method has been proven to be a good adjunct to pathological examination.

In 2006, Hadad and Bassagasteguy described a method using a mucoperiosteal NSF based on the nasoseptal artery, which is a branch of the posterior septal artery [8]. With a robust blood supply, large surface area, and broad arc of rotation, NSF has long been described as the workhorse pedicled flap for a wide variety of skull base defects. Although its use has been widely described as a reconstructive method for skull base defects, its utility in postirradiated skull base ORN has not yet been reported and advocated. In this case, the treatment of ORN using sequestrectomy and an NSF has demonstrated early satisfactory results, as compared with the traditional way in which defects were treated by secondary healing. The wound healed approximately 2 weeks after surgery, as compared with our previous management with which the healing process usually took 2–3 months [2], and a 1-year follow-up of the patient was uneventful. NSF reconstruction has the advantage of providing immediate mucosal coverage with a vascularized pedicle flap, with improved hygiene of the nasopharynx and adjacent skull base and by possibly impeding the inflammatory process in the skull base. To the best of our knowledge, the utility of NSF in such an indication has not been previously reported.

## Conclusion

Skull base ORN remains one of the most challenging complications in patients with postirradiated NPC. Endoscopic sequestrectomy has been advocated as an approach for the treatment of skull base ORN. We presented our experience using an NSF for the reconstruction of resultant skull base defects after sequestrectomy. To our knowledge, this is the first case in medical literature to describe NSF usage in such an indication. Using an NSF for the reconstruction of resultant skull base defects might provide a better treatment outcome for skull base ORN in patients with postirradiated NPC.

## Consent

Written informed consent was obtained from the patient for publication of this Case report and any accompanying images. A copy of the written consent is available for review by the Editor-in-Chief of this journal.

## Ethical approval

This work was approved by the Institutional Review Board of Chang Gung Memorial Hospital.

## Abbreviations

NPC: Nasopharyngeal carcinoma
NSF: Nasoseptal flap
ORN: Osteoradionecrosis
